# Imaging epilepsy in larval zebrafish

**DOI:** 10.1016/j.ejpn.2020.01.006

**Published:** 2020-01

**Authors:** D.R.W. Burrows, É. Samarut, J. Liu, S.C. Baraban, M.P. Richardson, M.P. Meyer, R.E. Rosch

**Affiliations:** a MRC Centre for Neurodevelopmental Disorders, Institute of Psychiatry, Psychology and Neuroscience, King's College London, London, UK; b Department of Neurosciences, Research Center of the University of Montreal Hospital Center, Montreal, Quebec, Canada; c Department of Neurological Surgery and Weill Institute for Neuroscience, University of California, San Francisco, San Francisco, CA, USA; d Department of Basic and Clinical Neuroscience, Institute of Psychiatry, Psychology and Neuroscience, King's College London, London, UK; e Centre for Developmental Neurobiology, Institute of Psychiatry, Psychology and Neuroscience, King's College London, London, UK; f Department of Bioengineering, University of Pennsylvania, Philadelphia, PA, USA; g Department of Paediatric Neurology, Great Ormond Street Hospital for Children NHS Foundation Trust, London, UK

**Keywords:** Zebrafish, Calcium imaging, Dynamical systems, Genetic epilepsies, Epilepsy

## Abstract

Our understanding of the genetic aetiology of paediatric epilepsies has grown substantially over the last decade. However, in order to translate improved diagnostics to personalised treatments, there is an urgent need to link molecular pathophysiology in epilepsy to whole-brain dynamics in seizures. Zebrafish have emerged as a promising new animal model for epileptic seizure disorders, with particular relevance for genetic and developmental epilepsies. As a novel model organism for epilepsy research they combine key advantages: the small size of larval zebrafish allows high throughput *in vivo* experiments; the availability of advanced genetic tools allows targeted modification to model specific human genetic disorders (including genetic epilepsies) in a vertebrate system; and optical access to the entire central nervous system has provided the basis for advanced microscopy technologies to image structure and function in the intact larval zebrafish brain.

There is a growing body of literature describing and characterising features of epileptic seizures and epilepsy in larval zebrafish. Recently genetically encoded calcium indicators have been used to investigate the neurobiological basis of these seizures with light microscopy. This approach offers a unique window into the multiscale dynamics of epileptic seizures, capturing both whole-brain dynamics and single-cell behaviour concurrently. At the same time, linking observations made using calcium imaging in the larval zebrafish brain back to an understanding of epileptic seizures largely derived from cortical electrophysiological recordings in human patients and mammalian animal models is non-trivial.

In this review we briefly illustrate the state of the art of epilepsy research in zebrafish with particular focus on calcium imaging of epileptic seizures in the larval zebrafish. We illustrate the utility of a dynamic systems perspective on the epileptic brain for providing a principled approach to linking observations across species and identifying those features of brain dynamics that are most relevant to epilepsy. In the following section we survey the literature for imaging features associated with epilepsy and epileptic seizures and link these to observations made from humans and other more traditional animal models. We conclude by identifying the key challenges still facing epilepsy research in the larval zebrafish and indicate strategies for future research to address these and integrate more directly with the themes and questions that emerge from investigating epilepsy in other model systems and human patients.

## 1 Introduction

Genomic diagnostics have revolutionised our understanding of the aetiology of paediatric epilepsies – we can now identify gene mutations in a majority of certain patient cohorts [[1], [2], [3]]. This has promising implications for the future of precisely targeted treatments [4]. However, to effectively translate these insights into new treatments, a better understanding of the relationship of molecular cause and whole-brain dynamics in epilepsy is urgently needed. Currently it is not clear how dysfunction in a diverse range of biological pathways affected by epilepsy-associated gene mutations [5] converges to a fairly stereotyped set of epileptic clinical phenotypes [6]. Zebrafish may help bridge the gap between molecular genetics, theoretical models and observations made in clinical epilepsy research.

In the last decade, zebrafish have emerged as a leading vertebrate model to investigate early patterning and development [7,8]. The zebrafish has a complex, yet easily accessible central nervous system which has played a key role in investigating the pathophysiology of many human neurodevelopmental disorders ranging from autism to epilepsy [9,10]; C [[11], [12], [13], [14]]. Although it underwent a whole genome duplication, the zebrafish genome is highly homologous to the human genome, with over 80% conservation of disease-causing genes, whilst also being genetically tractable. Genome editing techniques such as CRISPR/cas9 enable the targeted mutation of genes implicated in human epilepsies and in combination with transposon-mediated integration now allow for the efficient generation of stable zebrafish transgenic lines potentially encompassing the broad range of genes implicated in epilepsy [15]. Zebrafish already play a crucial role in drug discovery [16]. Larval epileptic zebrafish have been used in “high-throughput” experiments, as a combination of simple behavioural locomotion read-outs coupled with electrophysiology measures led to the identification of several lead compounds for Dravet syndrome [9,17]. A recent review of this literature suggests that zebrafish models of epilepsy featuring spontaneous seizures may be more reliable in terms of clinical relevance and pharmacological predictability than their mammalian counterparts [18].

**Fig. 1:**
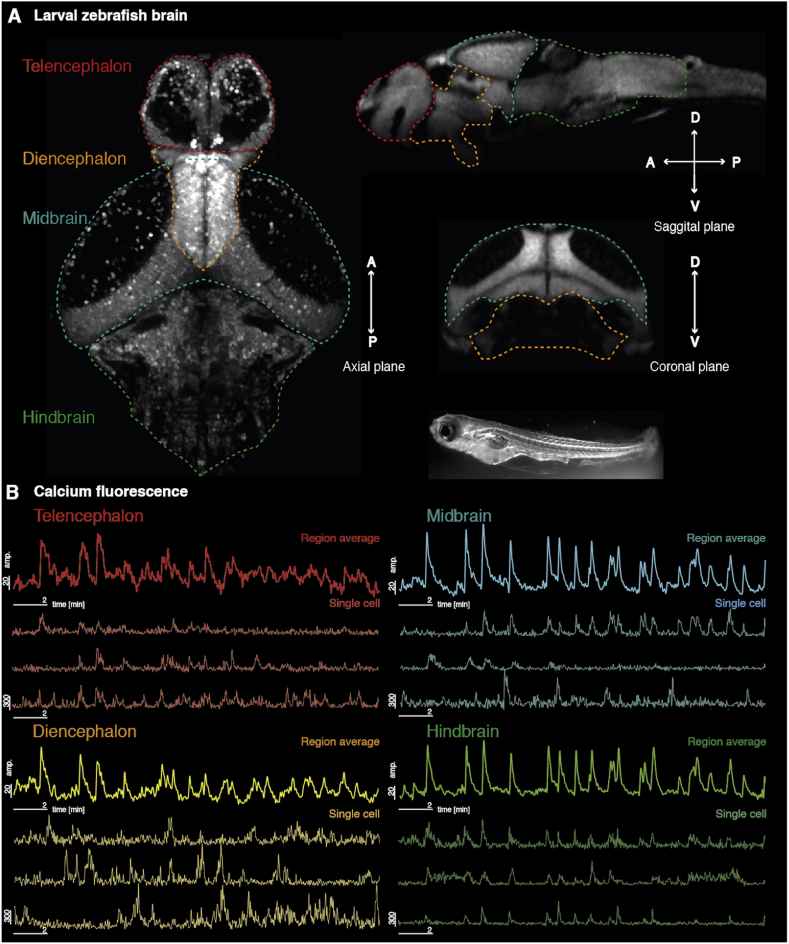
**Calcium imaging of larval zebrafish. (A)** A single axial plane from live two-photon imaging of a larval zebrafish brain is shown at 7 days post fertilisation on the left. On the right, sagittal and coronal views of a reference image [http://www.zbbrowser.com] [90] are shown to illustrate the 3D location of different brain areas. A zebrafish larva (not to scale) is shown at the bottom of the panel. **(B)** Example time series data are shown for regional averages of gross anatomical brain regions, as well as example cells identified in each area.

The external fertilisation in fish makes the embryo suitable for observations of pathological processes from the earliest stages of development [[19], [20], [21]]. At one week post fertilisation the zebrafish brain contains only ∼100,000 neurons (about one millionth of the human brain) and is < 1 mm^3^ in volume, yet displays canonical vertebrate brain anatomical divisions with high functional homology across brain regions to mammalian counterparts (Fig. 1A) [22,23]. The transparency of the embryo allows direct visualisation of central nervous system development with light microscopy tools [24]. With recent developments in microscopy this can now be achieved at near whole-brain coverage and single cell resolution (Fig. 1B) [19,25]. Furthermore, fluorescent transgenic calcium reporters such as GCaMP and RGECO [[26], [27], [28]] now facilitate live imaging of single neuron function during behaviour or at rest [19,29,30]. Thus, optical recordings of neuronal function allow for unprecedented spatial resolution (compared to local field potential, or electroencephalography recordings), with still considerable coverage (compared to single-cell electrophysiological recordings) - ideal for the investigation of epileptic dynamics, where both abnormal single cell behaviour, and population dynamics have been described [31,32].

Taken together - genetic tractability, small larval size, and availability of microscopy tools - zebrafish provide a unique opportunity to investigate neural circuitry underlying whole brain network dynamics in developmental disorders with genetic causes, including a range of epilepsies. Yet, much of our current understanding of epilepsy and epileptic seizures comes from electrophysiological recordings in the mammalian brain. Thus assessing the potential signatures of epileptic dynamics in the calcium imaging of the larval zebrafish brain, and integrating new insights with existing understanding of seizure dynamics provides new challenges. This review will introduce the reader to the state of the art of epilepsy research in zebrafish. We will review some key principles of epileptic dynamics and how they might manifest in calcium imaging of the epileptic brain. We will then illustrate promising data that is already available today in zebrafish models of epilepsy. The review then concludes with the key issues that need to be addressed in epilepsy imaging in zebrafish.

## 2 Zebrafish models of acute seizures and epilepsy

Since an initial publication by Baraban et al., in 2005, interest in zebrafish seizure models have grown substantially [33]. This pioneering study established protocols to monitor seizure behaviors and electrographic activity in larval zebrafish exposed to pentylenetetrazole (PTZ), a common convulsant agent. Multi-channel EEG recordings of adult zebrafish during PTZ induced seizures have shown qualitative homologies to human EEG recordings, with ictal discharge frequency (2–7 Hz) broadly comparable to scalp EEG in patients [34]. However, both EEG and LFP recordings in zebrafish have failed to demonstrate the presence of high gamma oscillations (80–150 Hz) shown with microelectrode array recordings in human and rodent (Weiss, S., et al., 2013), potentially due to the technical limitations of the recordings reported to date. Ongoing activity at rest in the zebrafish brain is quite distinct from mammalian activity - particularly in the larval brain, where activity is discontinuous and characterised by isolated bursts of activity [35]. In view of these differences, it is important to note that electrographic signatures of epileptic seizures are likely to be different across species and will require further detailed characterisation with the emergence of novel genetic models of epilepsy in fish.

Subsequent studies demonstrated the robustness of epileptic seizure-like activity in larval and adult zebrafish in response to proconvulsant drugs [[36], [37], [38]]. At a behavioural level, bath exposure to PTZ or kainic acid leads to a general increase in swim activity and, at higher drug concentrations, whole-body convulsive movements that are rarely observed in untreated wild-type zebrafish. In electrophysiological recordings obtained from the forebrain or midbrain of agarose-immobilized larvae, clear examples of recurrent brief interictal-like and long duration multi-spike ictal-like discharges are observed with these acute drug manipulations or even hyperthermia [39]. PTZ-evoked seizures in larval zebrafish are sensitive to antiepileptic drugs (AEDs) such as benzodiazepines and valproate [33,40,41] matching their long established effectiveness in treating PTZ-evoked seizures in rodents [[42], [43], [44]]. As such, zebrafish models can recapitulate both behavioural and electrophysiological features, as well as the response to AEDs of acute seizures seen in the mammalian brain.

Although these acute seizure models brought new insights (described in more detail below), they do not capture several key features of epilepsy, such as spontaneously occurring seizures, neurodevelopmental comorbidities, and certain features of interictal brain dynamics. However, increasingly we can identify underlying genetic aetiologies for many patients with epilepsy - particularly in the paediatric epilepsies. These known genetic mechanisms now offer the opportunity to model epilepsy beyond acutely induced seizures in zebrafish. Early studies described zebrafish with transient morpholino-based gene knockdown. In particular, zebrafish models for *lgi1* [45], *kcnq3* [46]*, chd2* [47], *kcnj10a* [35], *stx1b* [48] and *scn1lab* [49]. More stable and, perhaps more convincing, genetic models were originally described by the Baraban laboratory based on random ENU (*N-ethyl-N-nitrosourea*) mutagenesis screens: *mib/ube3A* [50] and *scn1lab* [9,51]. Interestingly, *scn1lab*^*552−/-*^larvae exhibit spontaneous abnormal electrographic activity, motor hyperactivity and convulsive activities starting at 3 dpf and persisting until early fatality around 10–12 dpf. This model of Dravet Syndrome has been characterised at behavioural, electrophysiological, transcriptomic and metabolic levels [9,52,53] and has been used extensively for drug-screening purposes [17,18,54]. More recently, the emergence of novel targeted mutagenesis techniques such as CRISPR/Cas9 opens the door to the generation of stable genetic models targeting all possible human epilepsy single gene mutations. To date, loss-of-function mutant zebrafish lines have been generated for *stxbp1* [55], *aldh7a1* [56], *gabra1* [57] and *depdc5* [14].

Note should be made that during evolution of teleost fish there has been a whole-genome duplication, which results in the presence of duplicates of many genes in zebrafish. Duplicated genes may differ in expression patterns and functional properties, resulting in somewhat unpredictable effects of individual gene knockouts. Individual knock-out of each of the duplicated genes may have opposing effects (e.g. in the two zebrafish orthologues of the human epilepsy gene *STXBP1* [58]), but at times the duplication may be an advantage, as homozygous knock-outs of one of the duplicated genes apparently mirror the effect of a heterozygous loss of function mutation in humans - i.e. reproduce the effects of haploinsufficiency, as for example in the commonly studied *scn1lab* −/− fish. Whether the effects of human gene mutations can be reproduced in fish by mutations in only one of two duplicated genes therefore needs to be carefully assessed for each gene individually. Overall, however, the diversity of these models now allows us to identify convergent mechanisms of epileptogenesis in the developing brain in a range of pathologies.

## 3 Dynamic signatures of epilepsy in the brain

From a century of experience in using scalp electrophysiology diagnostically in human patients with epilepsy, a combination of features have emerged as key electroencephalography (EEG) signatures of the epileptic brain [59]: These include specific ‘epileptiform’ discharges between or during epileptic seizures (e.g. spike and wave discharges), characteristic changes in the shape and composition of rhythmic EEG activity during the epileptic seizure that is usually identified by expert visual analysis (e.g. the epileptic recruiting rhythm at seizure onset), and some more subtle quantitative differences in EEG signal composition that can be measured through computational tools (e.g. increase in phase-locked high gamma activity [60], increased low alpha power [61], or increase low alpha phase synchrony [62]). Identifying possible calcium-imaging measures that may correspond to EEG markers of the epileptic brain is non-trivial because of the differences in spatial and temporal scales in these recording modalities: Imaging neuronal activity with genetically encoded calcium indicators has a much lower temporal resolution than electrophysiological recordings, yet affords separation of the signal of individual cells based on spatiotemporal firing properties [19,26].

**Fig. 2:**
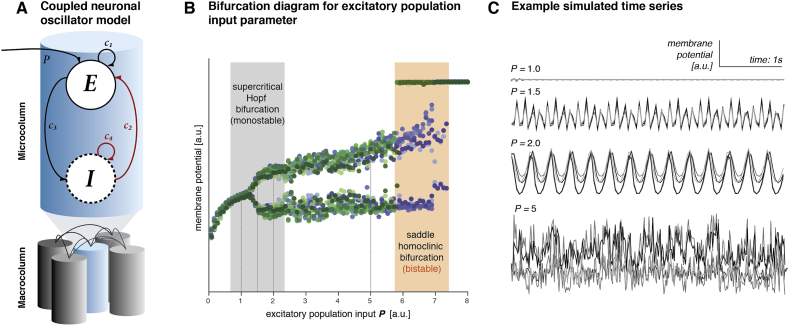
**Example of bifurcation behaviour of a set of coupled oscillators.** (A) Multiple Wilson-Cowan type neural mass models of excitatory and inhibitory populations were heterogeneously coupled and simulated at different values of excitatory population input *P* [112]. (B) This graph shows the membrane potential for five coupled microcolumns at steady state for different values of excitatory population input *P*, with dots of different shades representing a single microcolumn. Stepwise changes in *P* cause transitions in dynamics from fixed point steady states (shown as single dot per value *P*) to oscillatory states (shown as peak and trough of the oscillation for each value of *P* for *P* > 1.3) and back to fixed point (for *P* > 7.4). Simulations were run in small increasing (blue), and decreasing (green) steps, revealing bistability in the offset of the oscillation (note that outside of this bistability blue and green are largely overlapping). Even in this simplistic model many different state transition phenomena can be modelled, as sudden switches between high amplitude oscillations and fixed points at the bistable offset bifurcation, where two possible steady states co-exist. (C) Time series examples are shown for increasing values of the parameter *P* for illustration of different dynamic regimes associated with changes in just the single parameter *P*. (For interpretation of the references to colour in this figure legend, the reader is referred to the Web version of this article.)

Mathematical descriptions of dynamic systems may provide a conceptual bridge to explain phenomena and features of neuronal dynamics observed in different recording modalities [[63], [64], [65]]. Within the framework of dynamical systems, time-varying phenomena (such as neuronal firing or oscillations in an EEG recording) can be described using coupled differential equations that capture the evolution of a system's state in time [66]. Such descriptions are frequently applied to explain the dynamics of neuronal systems across multiple scales - ranging from descriptions of subcellular neuronal compartments [67] to whole-brain dynamics [[68], [69], [70]]. Even weak non-linearities in the behaviour of individual parts allow for the emergence of complex dynamics in the coupled systems (Fig. 2), including transitions between stable states driven by small changes in the underlying model parameters [71].

The key feature of the epileptic brain - i.e. the propensity to undergo epileptic seizures - can be elegantly captured in a dynamical system that undergoes bifurcations between different oscillatory regimes, for which there is now a large body of literature [6,[72], [73], [74]]. In fact, recent theoretical work has highlighted that a diverse range of seizure onset and offset patterns can be classified across species (including larval zebrafish) and seizure types using a small set of canonical bifurcations (some of which are shown in Fig. 2) [6]. These different bifurcations, in turn, can be reproduced in a simple neuronal oscillator model whose evolution over time resembles local field potential and EEG recordings of epileptic seizures [75,76].

Model based descriptions like these, help link a set of empirical observations to possible epilepsy-related seizure mechanisms. The specifics of these models can range from biophysically plausible models representing synaptic physiology [[77], [78], [79]], to more abstract network models restricted to capturing key interactions between regions [80,81]. These can be used to not only describe brain dynamics during an epileptic seizure, but also abnormal dynamics observed between seizures (i.e. interictal abnormalities) [62,65,82], and quantitative markers of dynamic states potentially associated with an increased propensity for epileptic seizure activity [[83], [84], [85]]. In Box 1 we illustrate how different features identified from models of dynamic systems may be identified in EEG and in calcium imaging data, respectively.

Box 1 **Features of abnormal brain dynamics in the epileptic brain and potential corresponding data features.**

**Table:** 

**Epileptic seizures**
● **Dynamic feature:** Epileptic seizures represent transitions between different steady states in coupled neuronal oscillator systems [6,90]. ● **EEG:** State changes might be visible as changes in the amplitude and frequency composition of ongoing oscillations, and between-region changes in signal correlation [91]. ● **Calcium imaging:** With the slower temporal resolution some shifts in high frequency bands may be missed, or – because of temporal summation – become apparent as increase in signal amplitude. Fluctuations of between-region or between-neuron correlations may also track state changes [86,[92], [93], [94], [95]].
**Interictal epileptiform discharges**
● **Dynamic feature:** The epileptic brain may show short-lived self-terminating transients that resembles features of epileptic seizures, even in between seizures. These may represent ‘ghosts’ of nearby bifurcations, and can increase with impending transitions between states [96]. ● **EEG:** Interictal discharges are well described and a key diagnostic criterion in human (and mammalian) EEG. Classification is usually based on morphology of the waveform and particular high- and low-frequency components (e.g. spike-and-wave) [97]. ● **Calcium imaging:** There is not yet a universally agreed classification of ‘epileptiform’ transients in calcium imaging. Future research may show the presence of short transients without behavioural correlate that share key features with epileptic seizure dynamics as candidate interictal discharge data features in calcium imaging.
**Markers of seizure propensity**
● **Dynamic feature:** Features of network architecture, intrinsic dynamics, or neuronal behaviour may render a network more prone to abnormal dynamic states and thus provide a mechanistic link between the makeup of the epileptic brain and its propensity for seizures [98]. ● **EEG:** Functional network synchronisability may provide a measure for how prone a network is to undergo generalised seizures. Excitation/inhibition imbalance may be quantifiable through spectral analysis, and quantification of specific markers of critical brain dynamics [80]. ● **Calcium imaging**: Some network features may translate from those identified in EEG - particularly those where a scale-invariant distribution is expected (e.g. markers of criticality [99]. But future work will be required to evaluate these candidate measures and their association with seizure threshold.

Alt-text: Box 1

When images are acquired at high enough sampling frequencies, many of the insights and models derived from EEG data also apply to larval zebrafish calcium imaging. Calcium imaging measures and electrophysiology are generally well correlated [86,87], even though the slow probe decay time or probe interference with calcium dynamics [88] need consideration. Yet challenges remain when applying these types of modelling strategies to data that are acquired at slower sampling frequencies (e.g. for whole volume recordings). Furthermore, the approaches illustrated do not address the unique strength of calcium imaging - i.e. the possibility to consider single cell behaviour. However, the challenges in integrating single cell dynamics into any model are not only technical in nature (i.e. analysing across thousands of time series representing individual neurons), but also speak to a conceptual challenge: Epileptic dynamics emerge from the integrated behaviour of whole coupled systems. Whilst epileptic seizures are associated with changes in single cell behaviour, it is the changes of the dynamics at the mesoscale - involving local circuits or whole networks - that are diagnostic hallmark of epileptic seizure activity. These mesoscale dynamics can not be accurately predicted from observations of isolated single cell behaviour alone and are thus often considered emergent properties [89].

Many features described in this section have already been examined in calcium imaging of mammalian epileptic seizures, and observations made in zebrafish are consistent with some key features above. The next challenge for the field is a detailed examination of corresponding multiscale features that can now be recorded in zebrafish models of both acute epileptic seizures, and of developmental epilepsies more broadly.

## 4 Imaging neuronal dysfunction in the zebrafish brain

Various multiscale dynamical features reported in the epileptic brain can now be examined in calcium imaging data from the larval zebrafish. Here we discuss key features derived from calcium imaging in the zebrafish brain that are associated with epileptic seizures and describe how these can be harnessed to understand the pathobiology of epilepsy.

Numerous studies have used imaging approaches to characterise how the GABA-A receptor antagonist, Pentylenetetrazol (PTZ) induces acute seizures in larval zebrafish [86,87,93]. These studies have reported a variety of dynamical features which shed light on the mechanisms of seizure generation which we will summarise below. Given the acute chemoconvulsant model, most of these descriptions focus on ictal transitions (cf Box 1), with questions regarding imaging features relevant to interictal activity and epileptogenesis remaining to be addressed.

### 4.1 State transitions

**Fig. 3:**
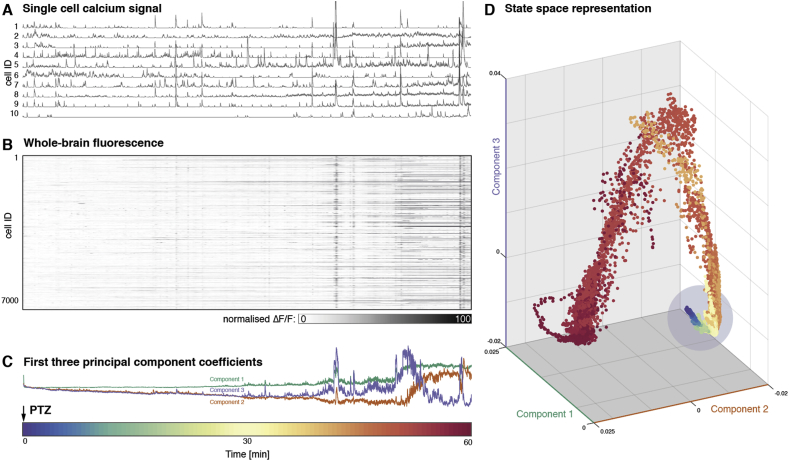
**Whole-brain state transition after PTZ exposure.** (A) Example normalised fluorescence traces are shown for individual neurons for 1 h after addition of PTZ to the bath at time point 0 showing an increase of amplitude and frequency of neuronal firing events. (B) Firing of all >7000 active cells captured in this recording. (C) First three principal components over time varying fluorescence matrix shown in (B). These indicate both a persistent drift in components 2, and 3, as well as drastic changes in the loading of all components towards the end of the recording. (D) The same data is shown as a state space plot. Whilst most of the data points exist in a restricted region/state (indicated by blue circle), the late seizure is characterised by very different activity distribution readily apparent in this low-dimensional projection as points outside of the earlier range. (Time scale for all figures shown as colour bar at the bottom of the figure). (For interpretation of the references to colour in this figure legend, the reader is referred to the Web version of this article.)

A key feature of epileptic seizures is the emergence of transitions from normal to abnormal brain states, often associated with abnormal neuronal activity, as well as abnormal synchronisation across neuronal networks [100]. Imaging of PTZ-induced seizures in zebrafish demonstrate changes in brain state that are characterised by aberrant levels of neuronal synchrony, following a characteristic time course. Across studies, these transitions into abnormal states are characterised by an increase in long-range (i.e. between gross anatomical brain regions) functional connectivity, suggesting a loss of the spatial constraints that normally bias connectivity towards nearby neurons and regions [86,87,93,95]. A concise way to represent such whole-brain state transitions is offered by examining time-varying changes in a low dimensional representation of either cellular activity, or time-varying functional connectivity in terms of principal components or related low-dimensional data projections [101]. Such approaches demonstrate excursions into abnormal state space following PTZ exposure, suggestive of seizure transition (Fig. 3). Whilst this makes identifying whole-brain transitions computationally possible, such approaches do not themselves offer mechanistic explanations for the transition. To elucidate the mechanisms underlying these state transitions we require an understanding of how microscale perturbations, at the level of cellular circuits, give rise to the macroscale network features of seizures.

### 4.2 Single cell activity

**Fig. 4:**
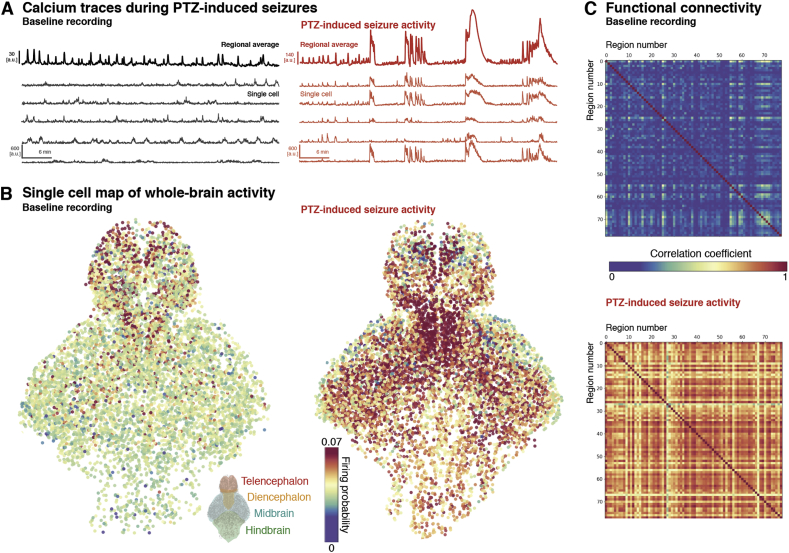
**Increases in neural whole-brain neuronal synchrony during PTZ-induced seizures.** (A) Example normalised fluorescence traces are shown as whole brain mean traces and examples of individual neurons at baseline (black) and PTZ-induced seizure (red) conditions (note the difference in amplitude scale). (B) Map of individual cell firing probabilities across the two conditions demonstrates an increase in firing probability in the PTZ-induced seizure condition, with regional heterogeneity across the larval fish brain. (C) Between-region functional connectivity shown as correlation matrix. Segmented cells were registered to a zebrafish brain atlas and then averaged, to measure correlation between major brain regions (>70) across baseline and PTZ conditions. (For interpretation of the references to colour in this figure legend, the reader is referred to the Web version of this article.)

A key and unique attribute of the larval zebrafish is that whole-brain imaging with single-neuron resolution can be used to examine how microscale changes at the level of individual neurons and local networks lead to the emergence of seizures at the macroscale. Seizure-like state transitions after PTZ administration, are characterised by sudden increases in neural synchrony which can be recorded both between individual neurons, and brain regions (Fig. 4) [87]. Detailed online atlases of the larval brain allow registration of imaging datasets to standard references, enabling identification of seizure networks with high anatomical specificity even across individual fish [102]. In this way, calcium imaging may link single neuron patterns of activity during seizures and whole brain dynamical states, which may provide a conceptual bridge for understanding the cellular pathophysiology underlying ictal phenomena typically recorded in EEG.

However, there is considerable heterogeneity of single neuron function within a network. Thus, to understand the cellular mechanisms underlying seizure state transitions, one requires an understanding of the contribution of specific cell subpopulations. This challenge can be addressed using double transgenic larval zebrafish that express fluorescent, genetically-encoded reporters of neural activity throughout the brain, and spectrally distinct fluorescent labels in specific subpopulations of cells such as excitatory- or inhibitory neurons, or glia [[103], [104], [105]]. Indeed preliminary evidence utilising such approaches suggest that glia-neuron interactions may be involved in transitions to generalised seizures in larval zebrafish [87]. Furthermore, with the emergence of cell-specific fluorescent reporters, one can tease apart the role of excitatory and inhibitory coupled activity in driving seizure transitions, particularly in genetic epilepsies where differential impairment of inhibitory populations have been proposed.

It is important to note that PTZ antagonises GABA-A receptors throughout the CNS [106], and therefore PTZ-induced seizures may not faithfully recapitulate spontaneous seizures, which can affect distinct brain regions, neuromodulatory mechanisms and cell subpopulations [107,108]. Nonetheless, several FDA-approved AEDs were initially discovered in PTZ models suggesting some translational relevance to these acute models [17]. Future work in genetic models - building on the imaging expertise established in acute PTZ models - will allow us to probe whether different mechanisms are at play in different epilepsies, and how distinct mechanisms may converge to similar dynamic phenotypes.

### 4.3 Excitation-inhibition balance

Another feature of epileptic seizures is a supposed loss of balance between excitation and inhibition. A growing body of empirical evidence suggests that the healthy brain operates at a critical point, enabling the dynamic fluctuation between divergent brain states [[109], [110], [111]]. Electrographic recordings in mice cortical slices and whole brain imaging of the larval zebrafish also suggest that levels of excitation and inhibition are poised at a specific balance, the ‘critical’ point that allows large-scale neuronal ‘avalanches’ that are rare and self-terminate thereby limiting runaway excitation [99]. Interestingly, in the epileptic brain the balance may be tipped toward a supercritical state in which activity rapidly maintains large avalanches of activity, i.e. a seizure [112]. Many features described in systems poised at this critical transition point exhibit scale-free behaviours. Therefore, any deviations from this critical point should be conserved across the micro and macro-scale [113]. These features make the idea of measures of criticality in the brain a promising feature for picking up abnormal EI balance from calcium imaging data [114].

**Fig. 5:**
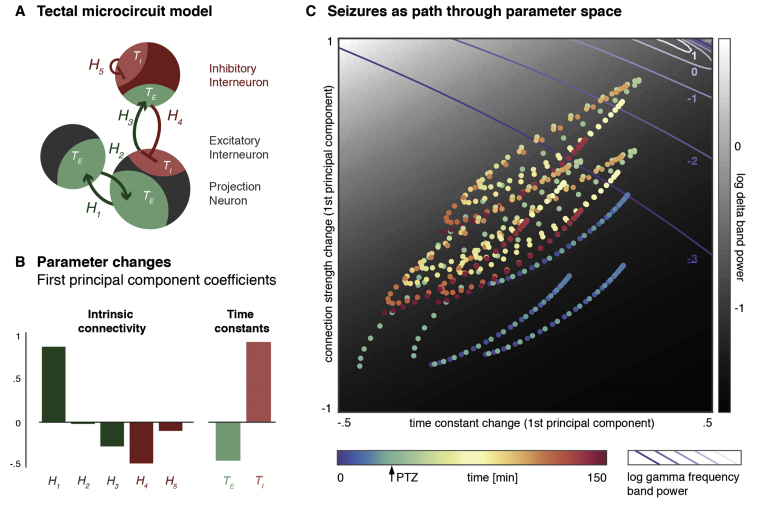
**Microcircuit model of tectal microcircuits during PTZ-induced epileptic seizures**. (A) Coupling parameters (time constants *T* and coupling strengths *H*) of a neuronal oscillator model were fitted to calcium imaging traces during induced epileptic seizures in larval zebrafish. (B–C) parameter values at different time points are plotted on two-dimensional projection. At each point of this two-dimensional space, model parameters can be used to simulate expected features of oscillatory (shown here in for delta-band oscillatory power as background grey scale map, and gamma-band oscillatory power as isoclines). This mapping between model parameters and predicted neuronal activity identifies key changes in parameters associated with PTZ exposure and seizure activity. Similar approaches may highlight regions in parameter space that are close to state transition points and render the brain more likely to display seizure activity. Figure reproduced with permission from Ref. [93].

Another strategy to link empirical observations with hypotheses on excitation-inhibition balance is provided by the use of specific mechanistic models. For example, Rosch et al. applied computational modelling to whole-brain functional imaging data from larval zebrafish to estimate the network-wide coupling parameters between recurrently coupled neuronal oscillators. This estimate was then used to determine the most likely changes in excitatory, and inhibitory neuronal coupling parameters that best explained the transition to a seizure [93], and in fact provide a full map of the parameter-dependent transitions between different dynamic regimes (Fig. 5). This approach, and other network modelling strategies may prove a useful tool to understand novel zebrafish models.

## 5 Future directions

Clinical epilepsy genetics, gene editing, and functional imaging have only very recently come together in a way that put zebrafish at the focal point of our research efforts in translating our understanding of the aetiology in paediatric epilepsy into understanding of pathomechanisms, and ultimately new treatments. In a relatively short time, across multiple different groups working in this space, remarkable progress has been made in developing innovative approaches to imaging seizures in larval zebrafish. Nonetheless, some challenges remain.

Single-gene epilepsies have emerged as a first important paradigm for further research into different childhood epilepsies. But the majority of presumed genetic epilepsies are likely caused by polygenic inheritance [2]. The recent use of zebrafish models of other polygenic neurodevelopmental disorders, such as schizophrenia, already illustrates how zebrafish may become useful for the study of these more prevalent polygenic epilepsies: First, their low cost allows generation of multiple knockout lines in parallel to identify possible converging phenotypic effects - most recently this has been done across >130 risk alleles associated with schizophrenia (Thyme et al., 2019).

Calcium imaging clearly allows for the identification of abnormal brain activity that carries hallmarks of epileptic discharges - such as hypersynchrony, and excessive amplitude. Whether or not a given abnormality in calcium imaging is in and of itself a seizure (ictal activity), or a mere marker of abnormal brain activity between seizures (interictal activity) remains challenging. In patient EEG, this distinction relies heavily on (a) the presence or absence of symptoms, and (b) the evolution of the abnormal activity in time, space, or frequency. In fish we will still need to develop a better understanding of normal behaviours during imaging, and normal evolution of large amplitude activity before clear classifications can be made with confidence. But combined behavioural and brain recordings will be one first step in that direction.

There is therefore a fundamental need to further develop the recording and analysis tools that help us delineate clearly epileptic from non-epileptic abnormalities in calcium imaging. This may at least in part be alleviated with improvements in imaging speed, and novel developments in voltage indicators [115]. Whilst some transitions are obvious even in low dimensional representations (e.g. Fig. 3), we risk missing important phenotypes by not being able to identify more subtle and short-lived abnormalities occurring spontaneously at unpredictable times. This is particularly relevant where seizures may be rare, as compared to long-term monitoring possible with electrophysiological recordings [116], the imaging duration is often limited by data storage, fish maintenance during the recording and bleaching of the fluorescence by the laser.

Yet it is precisely those phenotypes - paroxysmal, self-resolving abnormal dynamics with relatively normal brain function in between - that best represents the nature of epilepsy we encounter in most patients, and thus want to capture in our models. This need may be in part addressed by technological improvements (e.g. machine learning tools for classification of brain states, improved behavioural recording during imaging, etc). However, this also needs an improvement in the theory helping us make sense of this new type of data. What are the features that we expect at the different scales - recently calcium imaging of absence seizures in a mouse model for example suggested that neuronal synchrony may not be the whole answer [117]. Yet we do not currently have the theory readily available that specify cross-scale predictions that can be tested with our datasets. Once those improvements have been made, and new zebrafish models for the human genetic epilepsies in zebrafish emerge, we need to be able to develop the necessary pipelines that allow translation of these basic science insights into new therapies - both in the current drug screening approach, and in the targeted development of new treatments with image-guided insights into the pathophysiology that needs correcting.

As discussed, the two main benefits of using zebrafish models for human disease are their low-cost, their capacity for high-throughput screening, and the ease of gaining optical access to the entire central nervous system for calcium imaging. There are exciting emerging technological advances that allow the combination of neuroimaging and a medium-to-high throughput experimental setup through the use of microfluidic systems to automate larvae handling and imaging. Such systems have already been used to evaluate the effects of combinations of anti-seizure medication on normalising resting state and induced neuronal responses [118]. Some additional validation of these findings would be valuable, as the identified compounds differ from those identified in spontaneous seizure screens. Furthermore, the unpredictability and lower frequency of ictal-like events in spontaneous seizure models currently limits the use of imaging directly for drug screens. However, advances in data transfer speeds, robust preprocessing and online-analysis [119] that are currently being developed will likely allow for imaging to be combined with automatic sorting of larvae by functional network fingerprint, or seizure patterns in the near future.

## Declaration of competing interest

No other relevant affiliations or conflicts of interest are declared by any of the authors. ÉS a Co-Founder of Modelis inc. SCB is Co-Founder and Scientific Advisory Board member for Epygenix Therapeutics; and Scientific Advisory Board member for ZeClinics.
